# miRNA-584-3p inhibits gastric cancer progression by repressing Yin Yang 1- facilitated MMP-14 expression

**DOI:** 10.1038/s41598-017-09271-5

**Published:** 2017-08-21

**Authors:** Liduan Zheng, Yajun Chen, Lin Ye, Wanju Jiao, Huajie Song, Hong Mei, Dan Li, Feng Yang, Huanhuan Li, Kai Huang, Qiangsong Tong

**Affiliations:** 1 Department of Pathology, Union Hospital, Tongji Medical College, Huazhong University of Science and Technology, 1277 Jiefang Avenue, Wuhan 430022, Hubei Province P. R. China; 2 Clinical Center of Human Genomic Research, Union Hospital, Tongji Medical College, Huazhong University of Science and Technology, 1277 Jiefang Avenue, Wuhan 430022, Hubei Province P. R. China; 3 Department of Surgery, Union Hospital, Tongji Medical College, Huazhong University of Science and Technology, 1277 Jiefang Avenue, Wuhan 430022, Hubei Province P. R. China

## Abstract

Recent evidence shows the emerging roles of promoter-targeting endogenous microRNAs (miRNAs) in regulating gene transcription. However, miRNAs affecting the transcription of matrix metalloproteinase 14 (*MMP-14*) in gastric cancer remain unknown. Herein, through integrative mining of public datasets, we identified the adjacent targeting sites of Yin Yang 1 (YY1) and miRNA-584-3p (miR-584-3p) within *MMP-14* promoter. We demonstrated that YY1 directly targeted the *MMP-14* promoter to facilitate its expression in gastric cancer cells. In contrast, miR-584-3p recognized its complementary site within *MMP-14* promoter to suppress its expression. Mechanistically, miR-584-3p interacted with Argonaute 2 to recruit enhancer of zeste homolog 2 and euchromatic histone lysine methyltransferase 2, resulting in enrichment of repressive epigenetic markers and decreased binding of YY1 to *MMP-14* promoter. miR-584-3p inhibited the *in vitro* and *in vivo* tumorigenesis and aggressiveness of gastric cancer cells through repressing YY1-facilitated MMP-14 expression. In clinical gastric cancer tissues, the expression of YY1 and miR-584-3p was positively or negatively correlated with MMP-14 levels. In addition, miR-584-3p and YY1 were independent prognostic factors associated with favorable and unfavorable outcome of gastric cancer patients, respectively. These data demonstrate that miR-584-3p directly targets the *MMP-14* promoter to repress YY1-facilitated MMP-14 expression and inhibits the progression of gastric cancer.

## Introduction

Gastric cancer is the third leading cause of cancer-related death in the world^1^. Despite achievement in surgery and chemotherapy, the clinical outcome of patients with advanced gastric cancer still remains poor, mainly due to tumor growth and progression^1^. Therefore, it is an urgent duty to elucidate the mechanisms underlying the tumorigenesis and aggressiveness of gastric cancer^2^. Matrix metalloproteinase 14 (MMP-14), also known as membrane type-1 MMP, plays a pivotal role in digesting the extracellular matrice (ECM) and activating MMP-2, thereby promoting tumor invasion and metastasis^3^. MMP-14 also facilitates tumor angiogenesis via increasing the expression of vascular endothelial growth factor (VEGF)^4^ and releasing bioactive ECM products^3^. MMP-14 expression is elevated in most human cancers, and is associated with tumor invasion and metastasis^5^. It has been indicated that MMP-14 is highly expressed in gastric cancer and associated with poor survival of patients^6^, suggesting the importance of MMP-14 in the tumorigenesis and aggressiveness of gastric cancer.

Human *MMP-14* gene, consisting of 10 exons, locates at chromosome 14q11-12 and is regulated at the transcription level^7^. Previous studies have demonstrated that transcription factor early growth response 1 appears to be an essential regulator of *MMP-14* transcription in endothelial cells^8^, while specificity protein 1 is the critical factor for *MMP-14* promoter activity in fibrosarcoma cells^7^. The anti-tumorigenic effects of methylseleninic acid are mediated by the suppression of nuclear factor kappa B (NF-κB)-regulated MMP-14 levels in cancer cell lines^9^. In addition, MMP-14 is directly regulated by homeobox D10 in glioma^10^, proto-oncogene FBI-1 in ovarian cancer cells^11^, and hypoxia inducible factor 2 alpha in renal cancer^12^. However, the transcriptional regulators and underlying mechanisms essential for MMP-14 expression in gastric cancer still remain unclear.

In this study, through an integrative approach to mine public datasets, we identified Yin Yang 1 (YY1) and miRNA-584-3p (miR-584-3p) as crucial transcriptional regulators of MMP-14 expression in gastric cancer, with their adjacent targeting sites within the *MMP-14* promoter. We demonstrate, for the first time, that YY1 facilitates the expression of MMP-14 via directly binding to its promoter in gastric cancer. In contrast, miR-584-3p is down-regulated and negatively correlated with MMP-14 levels in clinical gastric cancer tissues, and directly targets the *MMP-14* promoter to inhibit its expression. Mechanistically, miR-584-3p interacts with Argonaute 2 (AGO2) to recruit enhancer of zeste homolog 2 (EZH2) and euchromatic histone lysine methyltransferase 2 (EHMT2), which results in enrichment of repressive epigenetic markers and decreased binding of YY1 to *MMP-14* promoter, thus inhibiting the tumorigenesis and aggressiveness of gastric cancer.

## Results

### YY1 facilitates the expression of MMP-14 in gastric cancer cells

**Figure 1:**
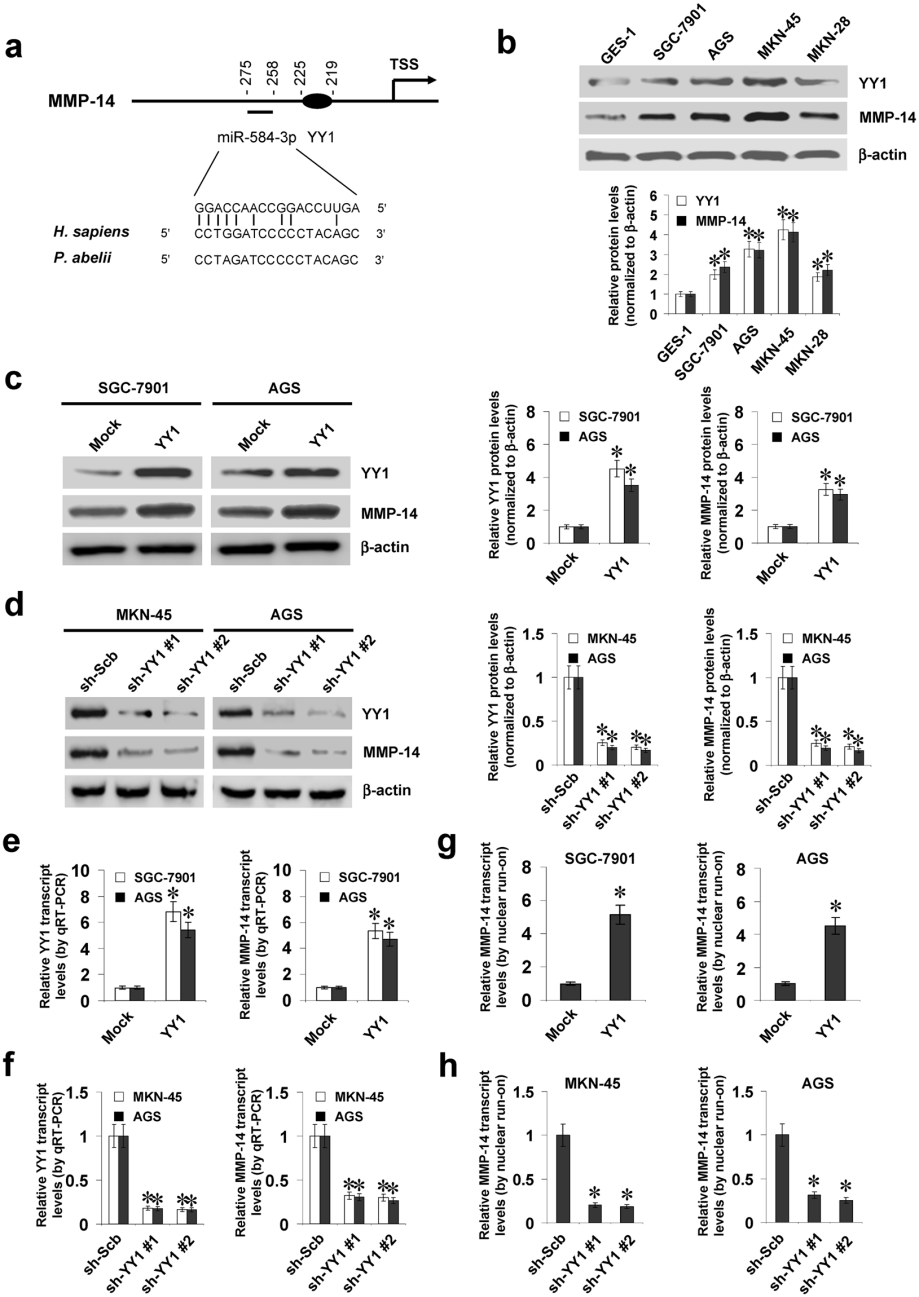
YY1 facilitates the expression of MMP-14 in gastric cancer cells. (**a**) Scheme of potential binding sites of YY1 and miR-584-3p within the *MMP-14* promoter, locating at bases −225/−219 and −167/−150 relative to TSS. (**b**) Western blot showing the expression of YY1 and MMP-14 in normal gastric epithelial GES-1 cells and gastric cancer cell lines SGC-7901, AGS, MKN-45, and MKN-28. (**c** and **d**) Western blot indicating the expression of YY1 and MMP-14 in SGC-7901, AGS and MKN-45 cells stably transfected with empty vector (mock), *YY1*, scramble shRNA (sh-Scb) or two *YY1* shRNAs (sh-YY1 #1 and sh-YY1 #2). (**e** and **f**) Real-time quantitative RT-PCR showing the transcript levels of *YY1* and *MMP-14* in gastric cancer cells stably transfected with mock, *YY1*, sh-Scb or sh-YY1 (mean ± SD, n = 5). (**g** and **h**) Nuclear run-on assay indicating the nascent *MMP-14* transcript levels in gastric cancer cells stably transfected with mock, *YY1*, sh-Scb, or sh-YY1 (mean ± SD, n = 4). *P < 0.01 vs. GES-1, mock, or sh-Scb.

To explore the mechanisms crucial for MMP-14 expression in gastric cancer, we analyzed the potential binding sites of transcription factors within its promoter using computational algorithm programs. Over-lapping analysis of Genomatrix^13^, TFBIND^14^, and PROMO^15^ revealed the potential binding sites of YY1, nuclear factor Y (NFY), and nuclear factor erythroid 2 like 2 (NFE2L2) within *MMP-14* promoter region (chr14:23304582-23305827; Supplementary Fig. S1a), locating at bases −225/−219, −158/−144, and −179/−159 upstream the transcription start site (TSS) of *MMP-14*, respectively. Further analysis of chromatin immunoprecipitation (ChIP) sequencing (ChIP-seq) data derived from UCSC Genome Browser^16^ revealed YY1 as the only enriched transcription factor within *MMP-14* promoter (Supplementary Fig. S1a). Meanwhile, the targeting site of miR-584-3p with high complementarity was noted at −275/−258 bp region surrounding that of YY1 (Fig. 1a). The expression levels of YY1 and MMP-14 were higher in gastric cancer cell lines, when compared to those in normal gastric epithelial cells (Fig. 1b). Notably, mining of publicly available Gene Expression Omnibus (GEO) datasets indicated that YY1 expression was positively correlated with MMP-14 levels in different gastric cancer cohorts (Supplementary Fig. S1b)^17–19^.

To address the hypothesis that YY1 might influence the MMP-14 expression, we performed the YY1 over-expression and knockdown experiments in gastric cancer cells. Stable transfection of *YY1* or two clones of short hairpin RNAs (shRNAs) targeting *YY1* (sh-YY1 #1 and sh-YY1 #2) into SGC-7901, AGS, and MKN-45 cells obviously up-regulated or decreased the protein and transcript levels of YY1 and MMP-14, than those stably transfected with empty vector (mock) or scramble short hairpin RNA (sh-Scb), respectively (Fig. 1c,d,e,f). Nuclear run-on assay demonstrated that stable over-expression or knockdown of *YY1* increased and decreased the nascent transcript levels of *MMP-14* in gastric cancer cells, respectively (Fig. 1g,h). In addition, the YY1-facilitated *MMP-14* transcription was not abolished by treatment with C646, the inhibitor of histone acetyltransferase (Supplementary Fig. S1c). These results suggested that YY1 facilitated the expression of MMP-14 in gastric cancer cells.

### YY1 directly binds to *MMP-14* promoter to increase its transcription

**Figure 2:**
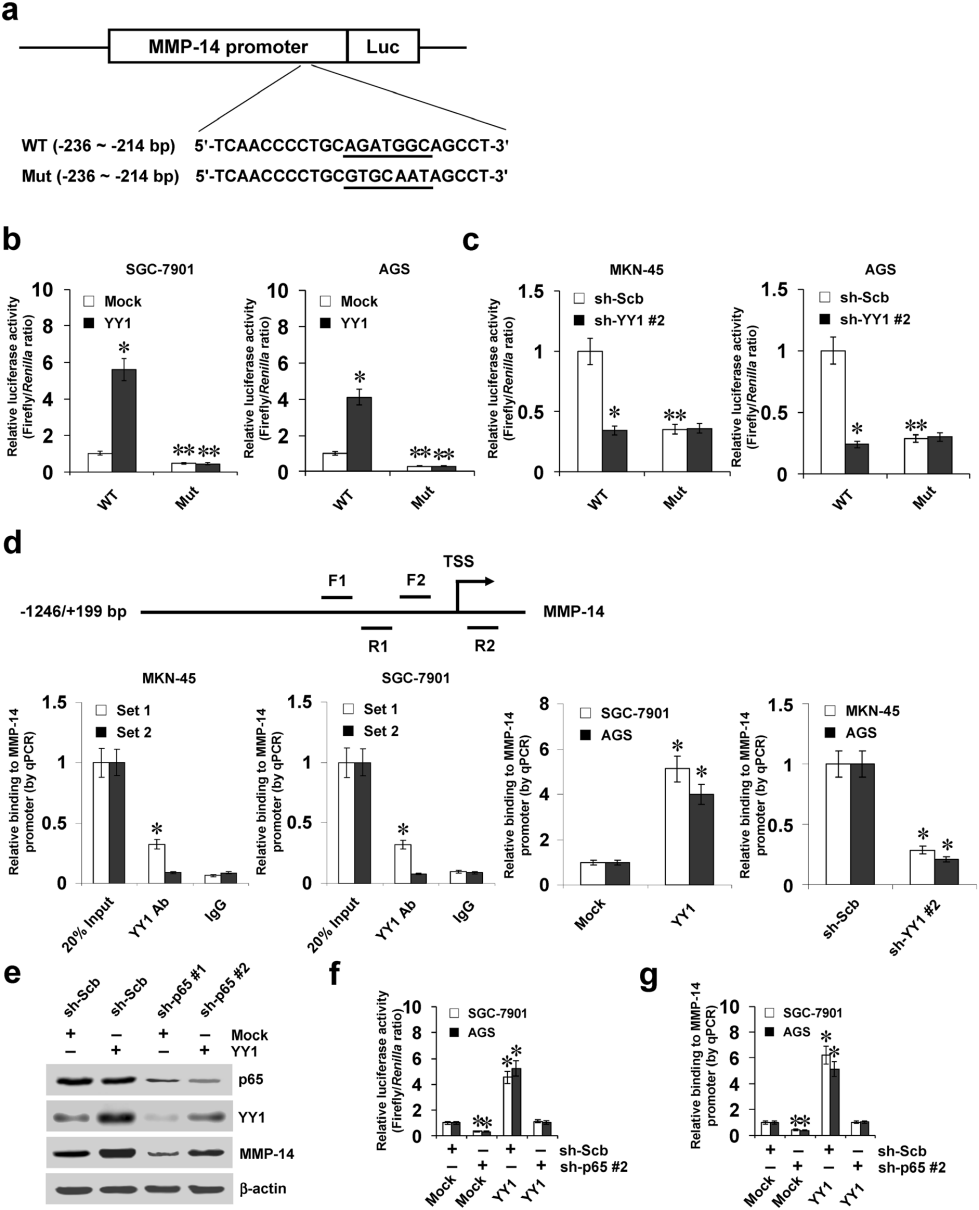
YY1 directly binds to *MMP-14* promoter to increase its transcription. (**a**) Scheme and sequence of the intact YY1 binding site (WT) and its mutation (Mut) within the *MMP-14* promoter-luciferase reporter vectors. (**b**) Dual-luciferase assay showing the activity (normalized to Mock + WT) of *MMP-14* promoter reporter [pGL3-MMP14 (-1246/ + 199)] and its mutant in SGC-7901 and AGS cells stably transfected with empty vector (mock) or *YY1* (mean ± SD, n = 5). (**c**) Dual-luciferase assay indicating the activity (normalized to sh-Scb + WT) of *MMP-14* promoter reporter [pGL3-MMP14 (−1246/+199)] and its mutant in MKN-45 and AGS cells stably transfected with scramble shRNA (sh-Scb) or *YY1* shRNA (sh-YY1) (mean ± SD, n = 4). (**d**) ChIP (using YY1 antibody) and qPCR assay showing the enrichment of YY1 (normalized to 20% input DNA) on the *MMP-14* promoter in gastric cancer cells, and those stably transfected with mock, *YY1*, sh-Scb, or sh-YY1 (mean ± SD, n = 5). (**e**) Western blot indicating the expression of p65, YY1, and MMP-14 in SGC-7901 cells transfected with mock or *YY1*, and those co-transfected with sh-Scb or sh-p65. (**f** and **g**) Dual-luciferase, ChIP (using YY1 antibody) and qPCR assays showing the activity of *MMP-14* promoter reporter [pGL3-MMP14 (-1246/ + 199)] and the binding of YY1 to *MMP-14* promoter in gastric cancer cells transfected with mock or *YY1*, and those co-transfected with sh-Scb or sh-p65 (mean ± SD, n = 5). *P < 0.01 vs. mock, sh-Scb, IgG, or mock + sh-Scb. **P < 0.01 vs. WT.

To investigate whether YY1 could target the *MMP-14* promoter to increase its transcription, the luciferase reporter and its mutation vectors of *MMP-14* promoter (Fig. 2a) were transfected into gastric cancer cells SGC-7901, AGS, and MKN-45. Dual-luciferase assay showed that ectopic expression or knockdown of *YY1* enhanced and attenuated the *MMP-14* promoter activity, respectively (Fig. 2b,c), and mutation of YY1 binding site abolished these effects (Fig. 2b,c). In addition, ChIP and real-time quantitative PCR (qPCR) indicated the enrichment of YY1 around its binding site (−326/−130 bp relative to TSS) in MKN-45 and SGC-7901 cells (Fig. 2d). As controls, no obvious *MMP-14* promoter regions were immunoprecipitated with unspecific antibody (isotype IgG) or detected by qPCR with primer set (−122/+69 bp) distal to the binding site of miR-584-3p (Fig. 2d). Stable over-expression of *YY1* resulted in enrichment of YY1 on the *MMP-14* promoter in gastric cancer cells (Fig. 2d). Meanwhile, stable knockdown of *YY1* with shRNA constructs decreased the binding of YY1 to *MMP-14* promoter in MKN-45 and AGS cells (Fig. 2d). Moreover, knockdown of *p65*, a subunit of NF-κB crucial for YY1 expression^20^, reduced the YY1 levels and prevented the gastric cancer cells from enhanced expression of YY1 and MMP-14, increased activity of *MMP-14* promoter, and increased binding of YY1 to *MMP-14* promoter induced by ectopic expression of *YY1* (Fig. 2e,f,g). These results indicated that YY1 directly bond to *MMP-14* promoter to enhance its transcription.

### miR-584-3p interacts with AGO2 to repress the YY1-facilitated *MMP-14* transcription

**Figure 3:**
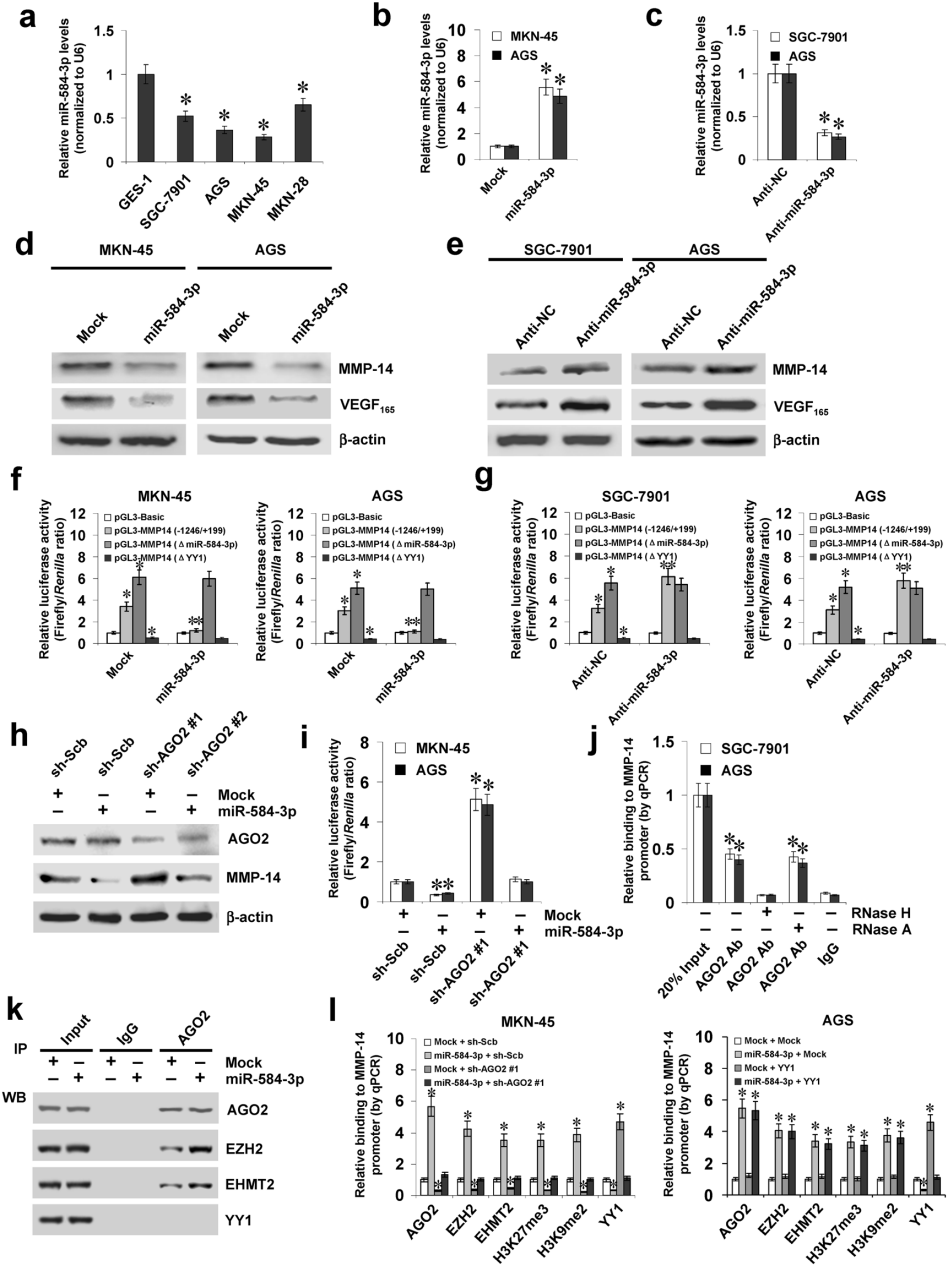
miR-584-3p interacts with AGO2 to repress the YY1-facilitated *MMP-14* transcription. **(a**) Real-time quantitative RT-PCR assay showing the expression of miR-584-3p in normal gastric epithelial GES-1 cells and gastric cancer cell lines SGC-7901, AGS, MKN-45, and MKN-28 (mean ± SD, n = 4). (**b** and **c**) Real-time quantitative RT-PCR assays indicating the expression of miR-584-3p in gastric cancer cells transfected with empty vector (mock), miR-584-3p precursor, negative control inhibitor (anti-NC, 100 nmol/L), or anti-miR-584-3p inhibitor (100 nmol/L) (mean ± SD, n = 4). (**d** and **e**) Western blot assay showing the expression of MMP-14 and VEGF_165_ in gastric cancer cells transfected with mock, miR-584-3p precursor, anti-NC (100 nmol/L), or anti-miR-584-3p inhibitor (100 nmol/L). (**f** and **g**) Dual-luciferase assay indicating the *MMP-14* promoter activity (normalized to pGL3-Basic) in gastric cancer cells transfected with mock, miR-584-3p precursor, anti-NC (100 nmol/L), or anti-miR-584-3p inhibitor (100 nmol/L; mean ± SD, n = 4). (**h** and **i**) Western blot and dual-luciferase assays showing the expression of AGO2 and MMP-14 and activity of *MMP-14* promoter reporter [pGL3-MMP14 (−1246/ + 199)] in gastric cancer cells stably transfected with mock or miR-584-3p precursor, and those co-transfected with scramble shRNA (sh-Scb) or *AGO2* shRNA (sh-AGO2). (**j**) ChIP (using AGO2 antibody) and qPCR assay indicating the binding of AGO2 (normalized to 20% input DNA) to *MMP-14* promoter in gastric cancer cells treated with RNase H or RNase A (mean ± SD, n = 4). (**k**) co-IP and western blot assays showing the interaction of AGO2 with EZH2, EHMT2, and YY1 in MKN-45 cells stably transfected with mock or miR-584-3p precursor, running under the same experimental conditions (full-length blots are presented in Supplementary Figure S7). (l) ChIP and qPCR assay indicating the enrichment (normalized to mock + sh-Scb or mock + mock) of AGO2, EZH2, EHMT2, H3K27me3, H3K9me2, and YY1 on *MMP-14* promoter in MKN-45 and AGS cells stably transfected with mock or miR-584-3p precursor, and those co-transfected with sh-Scb, sh-AGO2, mock, or YY1 (mean ± SD, n = 5). *P < 0.01 vs. GES-1, mock, anti-NC, pGL3-Basic, mock + sh-Scb, mock + mock, or IgG. **P < 0.01 vs. mock or anti-NC.

Since previous studies have indicated the roles of promoter-targeting miRNAs in regulating gene transcription^21, 22^, we further observed the effects of miR-584-3p on the expression of MMP-14 in gastric cancer cells. Lower miR-584-3p expression was observed in gastric cancer cell lines, when compared to that in normal gastric epithelial cells (Fig. 3a). The miR-584-3p precursor was stably transfected into MKN-45 and AGS cells, resulting in increased miR-584-3p levels (Fig. 3b). Meanwhile, transfection of anti-miR-584-3p inhibitor obviously decreased the miR-584-3p levels in SGC-7901 and AGS cells (Fig. 3c). In addition, over-expression or knockdown of miR-584-3p decreased and increased the protein and transcript levels of MMP-14 in gastric cancer cells, when compared to those transfected with empty vector (mock) or negative control (anti-NC) inhibitor, respectively (Fig. 3d,e, Supplementary Fig. S2a, and Fig. S2b). Notably, ectopic expression or knockdown of miR-584-3p reduced and enhanced the activity of *MMP-14* promoter, respectively, which was abolished by mutation of miR-584-3p or YY1 binding site (Fig. 3f,g). The expression of *VEGF*_165_, the most abundant VEGF isoform^4^ and MMP-14 downstream target gene in gastric cancer^23^, was significantly decreased or enhanced in miR-584-3p over-expressing and knockdown gastric cancer cells, respectively (Fig. 3d,e, Supplementary Fig. S2a and b). The analysis of microPIR database^24^ revealed no miR-584-3p binding site within *VEGF* promoter, ruling out the possible roles of miR-584-3p in directly suppressing the *VEGF* transcription. Since AGO2 is involved in miRNA-induced transcriptional repression^21, 22^, shRNAs specific for *AGO2* were transfected into MKN-45 and AGS cells. Knockdown of *AGO2* attenuated the transcriptional repression of *MMP-14* induced by ectopic expression of miR-584-3p in gastric cancer cells (Fig. 3h,i, Supplementary Fig. S2c). ChIP and real-time qPCR assay indicated that in cultured SGC-7901 and AGS cells, the enrichment of AGO2 on *MMP-14* promoter was observed at the region (−326/−130 bp) around the targeting site of miR-584-3p (Fig. 3j). In addition, treatment of gastric cancer cells with RNase H, but not with RNase A, abolished the enrichment of AGO2 on the *MMP-14* promoter in (Fig. 3j). RNA immunoprecipitation (RIP) and co-immunoprecipitation (co-IP) assays indicated that ectopic expression of miR-584-3p facilitated its interaction with AGO2, and increased the association of AGO2 with histone methyltransferases EZH2 and EHMT2 (Supplementary Fig. S2d and Fig. 3k). However, YY1 was not associated with this multi-protein complex (Fig. 3k). Over-expression of miR-584-3p increased the levels of histone H3 lysine 27 trimethylation (H3K27me3) and histone H3 lysine 9 dimethylation (H3K9me2), which were abolished by knockdown of *AGO2* or treatment with established specific inhibitors of EZH2 and EHMT2, GSK343^25^ and A-366^26^, in gastric cancer cells (Supplementary Fig. S2e). Stable over-expression of miR-584-3p resulted in increased binding of AGO2 and epigenetic markers EZH2, EHMT2, H3K27me3 and H3K9me2, and decreased binding of YY1 to *MMP-14* promoter in gastric cancer cells (Fig. 3l), which was attenuated by knockdown of *AGO2* (Fig. 3l). Meanwhile, ectopic expression of *YY1* did not affect the enrichment of AGO2, EZH2, EHMT2, H3K27me3, or H3K9me2 on *MMP-14* promoter (Fig. 3l). Moreover, treatment with GSK343 and A-366 prevented the gastric cancer cells from increased enrichment of H3K27me3 and H3K9me2 and decreased binding of YY1 to *MMP-14* promoter induced by miR-584-3p over-expression (Supplementary Fig. S2f). Collectively, these data suggested that miR-584-3p interacted with AGO2 to repress the YY1-facilitated *MMP-14* transcription in gastric cancer cells.

### miR-584-3p suppresses the tumorigenesis and aggressiveness of gastric cancer cells via repressing YY1-facilitated MMP-14 expression *in vitro*

**Figure 4:**
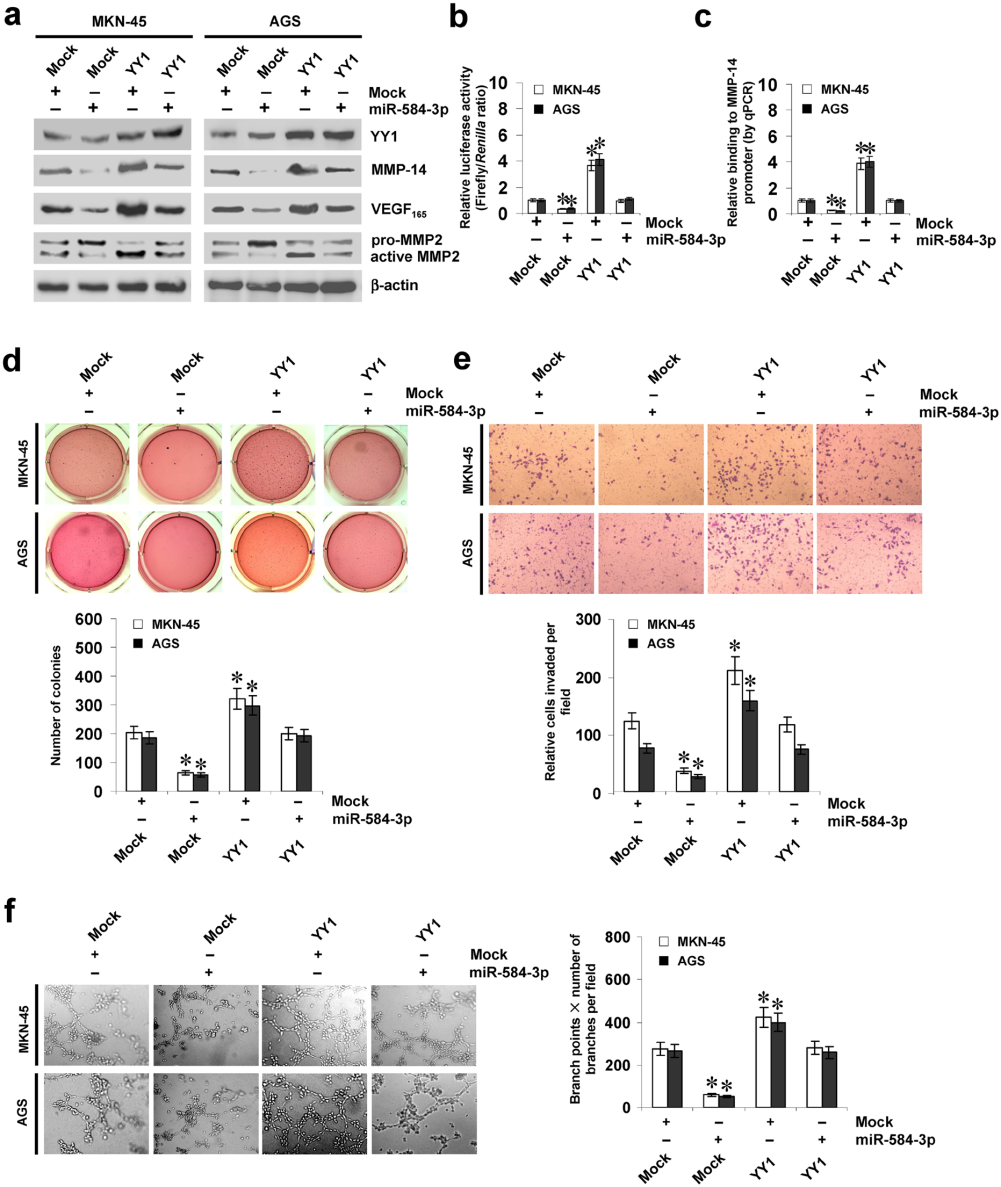
miR-584-3p suppresses the tumorigenesis and aggressiveness of gastric cancer cells via repressing YY1-facilitated MMP-14 expression *in vitro*. (**a** and **b**) Western blot and dual-luciferase assays showing the expression of YY1, MMP-14, VEGF_165_, and MMP-2 and activity of *MMP-14* promoter reporter [pGL3-MMP14 (-1246/ + 199)] in MKN-45 and AGS cells stably transfected with empty vector (mock) or miR-584-3p precursor, and those co-transfected with *YY1* (mean ± SD, n = 4). (**c**) ChIP (using YY1 antibody) and qPCR assay indicating the enrichment of YY1 on *MMP-14* promoter in gastric cancer cells stably transfected with mock or miR-584-3p precursor, and those co-transfected with *YY1* (mean ± SD, n = 4). (**d**) Representation (top) and quantification (bottom) of soft agar assay showing the anchorage-independent growth of MKN-45 and AGS cells stably transfected with mock or miR-584-3p precursor, and those co-transfected with *YY1* (mean ± SD, n = 5). (**e**) Representation (top) and quantification (bottom) of matrigel invasion assay indicating the invasion capability of gastric cancer cells stably transfected with mock or miR-584-3p precursor, and those co-transfected with *YY1* (mean ± SD, n = 5). (**f**) Representation (top) and quantification (bottom) of tube formation assay showing the angiogenic capability of gastric cancer cells stably transfected with mock or miR-584-3p precursor, and those co-transfected with *YY1* (mean ± SD, n = 5). *P < 0.01 vs. mock + mock.

Since above evidence indicated that miR-584-3p repressed the binding of YY1 to *MMP-14* promoter, we further investigated the effects of miR-584-3p over-expression on YY1-facilitated MMP-14 levels in cultured gastric cancer cells. Ectopic expression of miR-584-3p did not affect the YY1 expression levels (Fig. 4a), but abolished the enhanced protein and transcript levels of *MMP-14* and increased levels of VEGF_165_ and active MMP-2 induced by stable transfection of *YY1* (Fig. 4a,b, Supplementary Fig. S3a). In addition, over-expression of miR-584-3p prevented the enhanced YY1 enrichment on *MMP-14* promoter induced by ectopic expression of *YY1* (Fig. 4c). Notably, ectopic expression of miR-584-3p abolished the increase in expression and promoter activity of *MMP-14* and enrichment of YY1 induced by transfection of *YY1* in prostate cancer PC-3 cells and colon cancer LoVo cells (Supplementary Fig. S3b, Fig. S3c, and Fig. S3d), suggesting the potential roles of miR-558-3p and YY1 in regulating MMP-14 expression in these types of cancers. In soft agar assay, YY1 over-expression promoted the anchorage-independent growth of MKN-45 and AGS cells (Fig. 4d). In matrigel invasion assay, stable over-expression of YY1 increased the invasion capacity of gastric cancer cells (Fig. 4e). The tube formation of endothelial cells was increased by treatment with the medium preconditioned by gastric cancer cells stably transfected with *YY1* (Fig. 4f). Moreover, transfection of miR-584-3p rescued the MKN-45 and AGS cells from increased growth, invasion, and angiogenesis capability induced by stable over-expression of *YY1* (Fig. 4d,e,f). On the other hand, stable knockdown of *YY1* resulted in down-regulation of MMP-14 (Supplementary Fig. S4a) and decreased capability in growth (Supplementary Fig. S4b,c), invasion (Supplementary Fig. S4d), and angiogenesis (Supplementary Fig. S4e) in SGC-7901 and AGS cells. Down-regulation of miR-584-3p rescued the SGC-7901 and AGS cells from their changes in these biological features induced by knockdown of *YY1* (Supplementary Fig. S4,b,c,d,e). Meanwhile, ectopic expression or knockdown of *MMP-14* rescued the gastric cancer cells from alteration in the viability, invasion, and angiogenesis induced by over-expression of miR-584-3p or YY1, respectively (Supplementary Fig. S5). These results suggested that miR-584-3p decreased the tumorigenesis and aggressiveness of gastric cancer cells through repressing YY1-facilitated MMP-14 expression *in vitro*.

### miR-584-3p attenuates the YY1-facilitated growth, metastasis, and angiogenesis of gastric cancer cells *in vivo*

**Figure 5:**
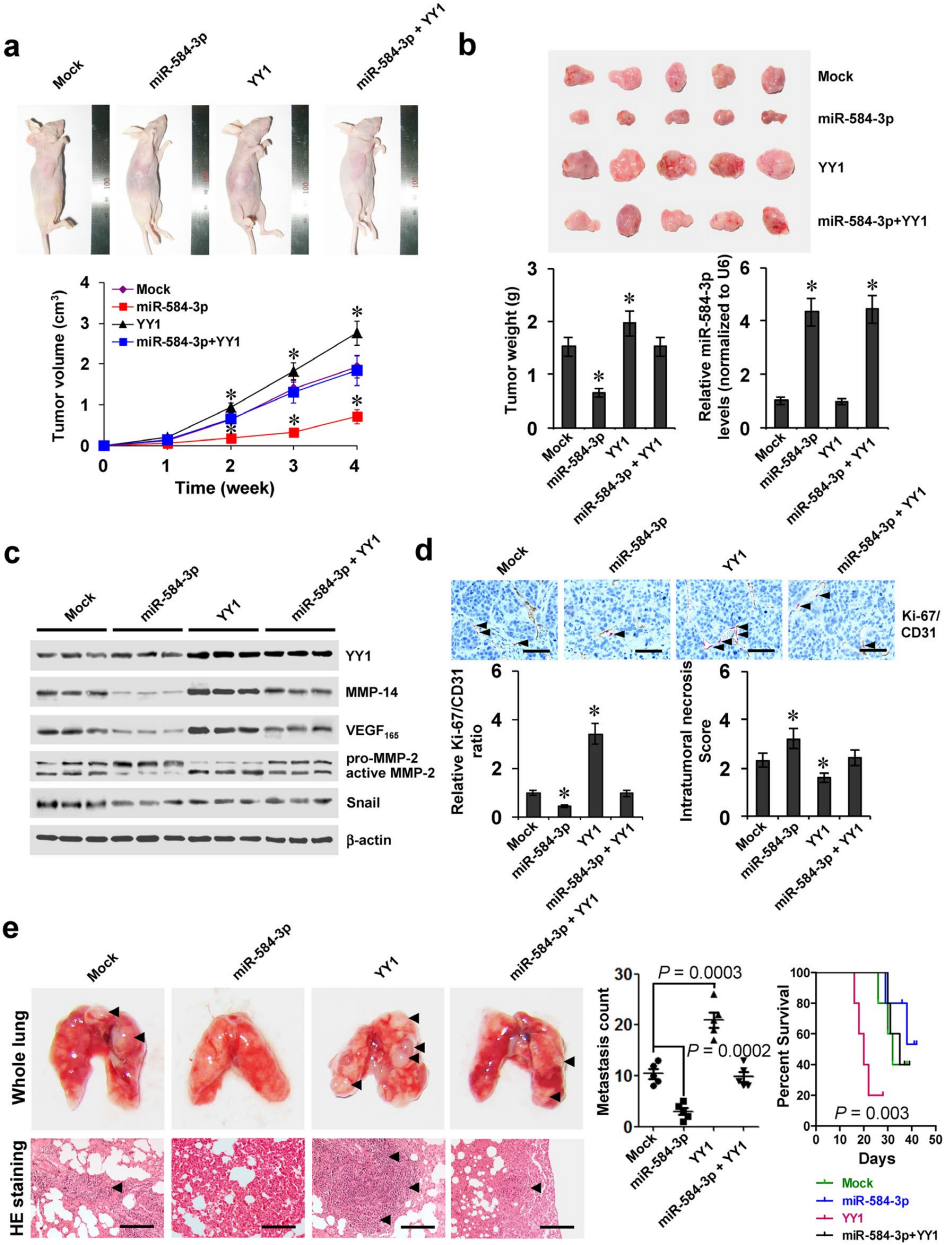
miR-584-3p attenuates the YY1-facilitated growth, metastasis, and angiogenesis of gastric cancer cells *in vivo*. (**a**) Tumor growth curve of SGC-7901 cells (1 × 10^6^) stably transfected with empty vector (mock) or miR-584-3p precursor, and those co-transfected with *YY1* in athymic nude mice (n = 5 for each group), after hypodermic injection for 4 weeks. (**b**) Representative images (top), weight (bottom), and miR-584-3p levels (bottom) of xenograft tumors formed by hypodermic injection of SGC-7901 cells stably transfected with mock or miR-584-3p precursor, and those co-transfected with *YY1* (mean ± SD, n = 5). (**c**) Western blot showing the expression of YY1, MMP-14, VEGF_165_, and MMP-2 within tumors formed by hypodermic injection of SGC-7901 cells stably transfected with mock or miR-584-3p precursor, and those co-transfected with *YY1* (mean ± SD, n = 5). (**d**) Double immunohistochemical staining (top) and quantification (bottom) of Ki-67 (red, arrowheads)/CD31 (brown) expression and necrosis area within tumors formed by hypodermic injection of SGC-7901 cells stably transfected with mock or miR-584-3p precursor, and those co-transfected with *YY1* (mean ± SD, n = 5). Scale bars: 100 μm. (**e**) Representation (left, arrowheads) and quantification (middle) of lung metastasis and Kaplan–Meier survival plots (right) of nude mice with injection of SGC-7901 cells (0.4 × 10^6^) stably transfected with mock or miR-584-3p precursor, and those co-transfected with *YY1* via the tail vein (n = 5 for each group). Scale bars: 100 μm. *P < 0.001 vs. mock.

We further explored the effects of miR-584-3p on YY1-facilitated tumor growth and metastasis *in vivo*. Stable over-expression of *YY1* led to increased *in vivo* growth of SGC-7901 cells in athymic nude mice and enhanced tumor weight of established subcutaneous xenograft tumors (Fig. 5a,b). The expression of YY1, MMP-14, VEGF_165_, active MMP-2, but not of Snail^27^, was enhanced in xenograft tumors formed by SGC-7901 cells stably transfected with *YY1* (Fig. 5c). In addition, stable transfection of *YY1* increased the Ki-67/CD31 ratio of microvessels within tumors and decreased the intratumoral necrosis area (Fig. 5d). Moreover, stable transfection of *YY1* into SGC-7901 cells established statistically more lung metastatic colonies and lower survival probability than empty vector (mock) group (Fig. 5e). Meanwhile, stable over-expression of miR-584-3p precursor led to increase in the miR-584-3p levels, and rescued the YY1-facilitated growth, gene expression, metastasis and angiogenesis of SGC-7901 cells in athymic nude mice (Fig. 5a,b,c,d,e). These data indicated that miR-584-3p could attenuate the YY1-facilitated growth, metastasis, and angiogenesis of gastric cancer cells *in vivo*.

### YY1 and miR-584-3p are positively or inversely correlated with MMP-14 levels in gastric cancer tissues

**Figure 6:**
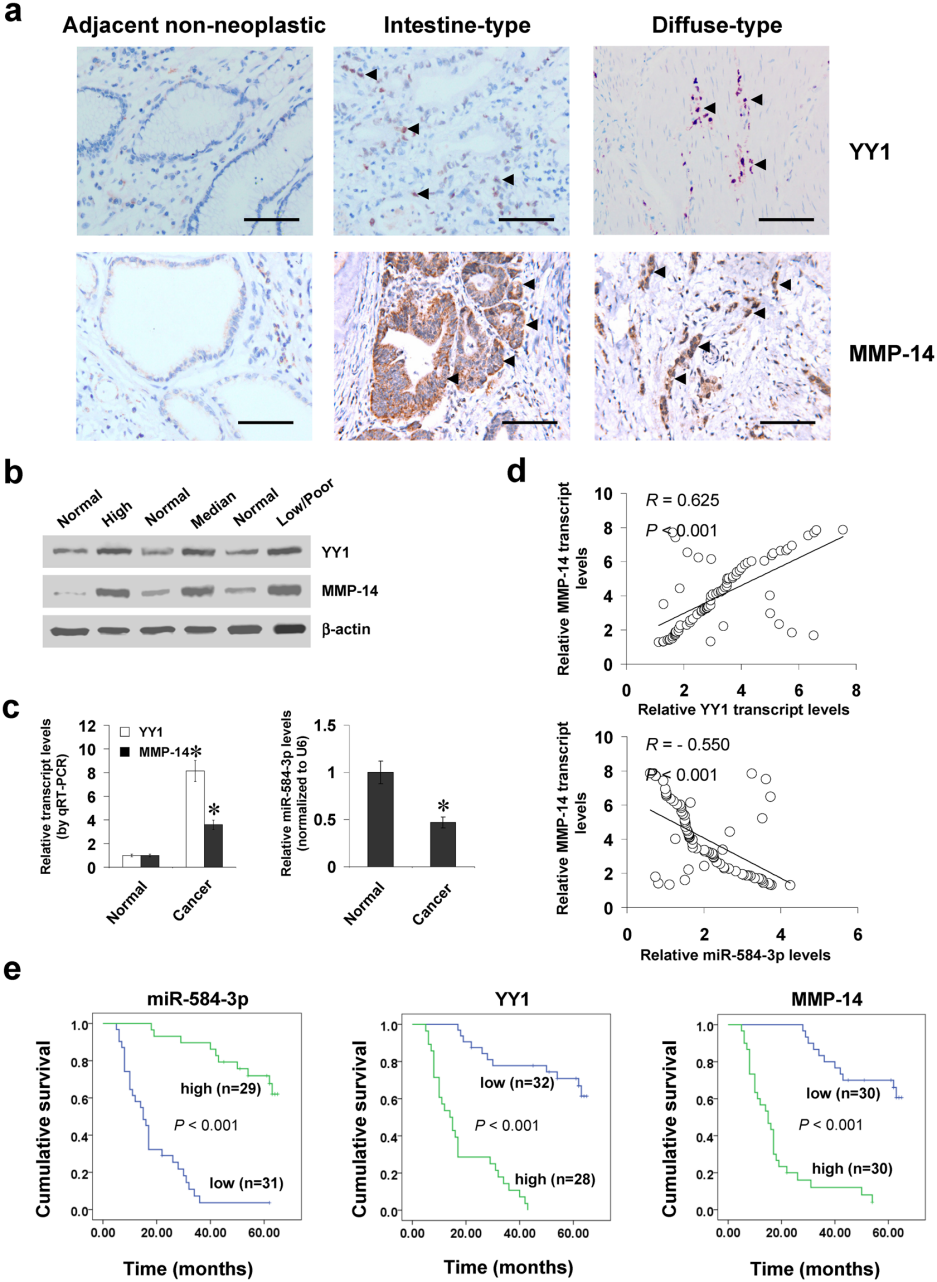
YY1 and miR-584-3p are positively or inversely correlated with MMP-14 levels in gastric cancer tissues. (**a**) Immunohistochemical staining showing the expression of YY1 and MMP-14 in the tumor cells of gastric cancer specimens (arrowheads, brown). Scale bars: 100 μm. (**b**) Western blot assay indicating the protein levels of YY1 and MMP-14 in gastric cancer tissues with different differentiation, and those in normal gastric mucosa. (**c**) Real-time quantitative RT-PCR showing the transcript levels of *YY1*, *MMP-14*, and miR-584-3p in normal gastric mucosa (n = 80) and gastric cancer tissues (n = 80). (**d**) The correlation between *MMP-14* transcript levels and *YY1* or miR-584-3p expression in gastric cancer tissues (n = 80). (**e**) Kaplan–Meier survival plots of 60 well-defined gastric cancer cases with high or low expression of miR-584-3p, YY1, or MMP-14. *P < 0.01 vs. normal.

To reveal the expression of YY1 in gastric cancer tissues, immunohistochemical staining was performed on paraffin-embedded sections from 60 well-established primary cases. The results indicated nuclear YY1 expression in cancer cells (Fig. 6a), which was detected in 36/60 (60.0%) cases, with weak staining in 10, moderate in 14, and intense in 12 (Supplementary Table S1). Higher YY1 expression was observed in gastric cancer cases with deeper gastric wall invasion (*P* < 0.001), lymph node metastasis (*P* < 0.001), distant metastasis (*P* = 0.005), or advanced tumor-node-metastasis (TNM) stage (*P* < 0.001) (Supplementary Table S1). Notably, there was positive correlation between YY1 and MMP-14 immunoreactivity in gastric cancer cases (correlation coefficient *R* = 0.802, *P* < 0.001, Fig. 6a and Supplementary Table S2). In 80 fresh gastric cancer specimens, higher protein and transcript levels of YY1 or MMP-14 were observed than those in normal gastric mucosa (Fig. 6b,c). In contrast, miR-584-3p was under-expressed in gastric cancer tissues than in normal gastric mucosa (Fig. 6c and Supplementary Fig. S6a)^28^. The levels of SH3 domain and tetratricopeptide repeats 2 (SH3TC2), the host gene of miR-584-3p^29^, were decreased in gastric cancer tissues derived from public datasets (Supplementary Fig. S6b)^19, 30^. A positive correlation between *YY1* and *MMP-14* transcript levels was noted in gastric cancer tissues (correlation coefficient *R* = 0.625, *P* < 0.001, Fig. 6d). Meanwhile, the miR-584-3p expression was inversely correlated with *MMP-14* transcript levels in gastric cancer tissues (*R* = −0.550, *P* < 0.001, Fig. 6d). The inverse correlation between SH3TC2 and MMP-14 was also noted in gastric cancer specimens derived from public datasets (Supplementary Fig. S6c)^18, 31, 32^. Kaplan–Meier survival analysis revealed that patients with low miR-584-3p levels (*P* < 0.001), high YY1 levels (*P* < 0.001), or high MMP-14 expression (*P* < 0.001) had lower survival probability (Fig. 6e). Cox regression analysis of these gastric cancer cases indicated that distant metastasis (hazard ratio *HR* = 2.126, *P* = 0.003), TNM stage (*HR* = 2.352, *P* = 0.001), miR-584-3p expression (*HR* = 0.312, *P* = 0.018), and YY1 levels (*HR* = 2.743, *P* = 0.011) were independent prognostic factors for gastric cancer patients (Supplementary Table S3). These results indicated the up-regulation of YY1 and under-expression of miR-584-3p in gastric cancer tissues, which were positively and inversely correlated with the MMP-14 levels, respectively.

## Discussion

YY1, one member of the GLI-Krüppel protein family, plays a fundamental role in embryogenesis, differentiation, chromosomal dynamics, X-chromosome inactivation, and DNA repair^33^. Recent evidence shows the oncogenic or tumor repressive roles of YY1 in tumorigenesis and aggressiveness^34, 35^. Ectopic expression of *YY1* drives the proliferation of cancer cells via increasing C-myc expression and decreasing P53 activity^34^. In addition, YY1 promotes the cell invasion, angiogenesis, and metastasis of osteosarcoma^36^, and its high expression in osteosarcoma tissues is associated with occurrence of metastasis and poor clinical outcome^37^. Depletion or knockdown of *YY1* significantly decreases the tumorigenesis and aggressiveness of bone cancer cells^36^. Enhanced expression of YY1 has been documented in many types of human malignancies^38^, and is associated with poor prognosis^36^. On the other hand, YY1 might exert tumor suppressive functions in certain types of cancers. High expression of YY1 predicts favorable outcome with longer survival in follicular lymphoma^35^. Previous studies have shown that YY1 is elevated in gastric cancer specimens, and knockdown of *YY1* inhibits the proliferation of gastric cancer cells *in vitro* ^39^. However, the roles and underlying mechanisms of YY1 in the tumorigenesis and aggressiveness of gastric cancer still remain largely unknown. In this study, our data showed that YY1 facilitated the *in vitro* and *in vivo* growth, metastasis, and angiogenesis of gastric cancer cells, and patients with high expression of YY1 had lower survival probability, indicating the oncogenic functions of YY1 in gastric cancer progression.

As a transcription factor, YY1 regulates gene transcription as an activator, initiator, or repressor in a cell type- and sequence context-dependent manner^38^. YY1 exerts its oncogenic property through activating the expression of c-Myc^40^ and epidermal growth factor receptor 2^41^. YY1 binds to the promoter of prostate stem cell antigen to facilitate the development of prostate cancer^42^. In melanoma cells, YY1 activates the transcription of *Snail* through binding to the 3’ enhancer, suggesting its involvement in the epithelial-mesenchymal transition during cancer metastasis^27^. In addition, YY1 also negatively regulates the expression of tumor suppressor genes p27, p16, and p73^38^. It has been indicated that as a transcription factor, YY1 binds to specific DNA sequence and directs the polycomb group (PRC) complexes to target loci, resulting in suppression of gene expression^43^. However, in this study, we found that YY1 was not associated with PRC complex for MMP-14 expression in gastric cancer cells. Our evidence indicated that YY1 expression was positively correlated with MMP-14 levels in gastric cancer specimens and cell lines. Importantly, YY1 directly bond to *MMP-14* promoter to increase its expression, resulting in elevated levels of VEGF and active MMP-2, but not of Snail, in gastric cancer cells. Previous studies have shown that NF-κB facilitates the expression of YY1 through the binding of p65/p50 subunits to *YY1* promoter^20^. We found that genetic ablation of the p65 subunit of NF-κB attenuated the expression of YY1 and downstream target MMP-14, shedding light on a potential approach to regulate the MMP-14 expression in gastric cancer.

In this study, we noted the adjacent binding sites of miR-584-3p and YY1 within *MMP-14* promoter. MiRNAs are a class of small non-coding RNAs that mainly target binding sites in 3’-untranslated regions, and interact with members of the AGO protein family to suppress translation or degrade mRNA^44^. Recent studies indicate that endogenous miRNAs can recognize complementary genomic sites within human gene promoters, and participate in the heterochromatin formation and regulation of gene transcription^21, 22^. For example, miR-423-5p recruits AGO2 to repress the transcription of progesterone receptor via inducing DNA methylation and H3K9me2 enrichment in breast cancer cells^21^. In human cells, let-7 forms complex with AGO2 to repress the transcription of retinoblastoma 1/E2F downstream genes in senescence^22^. On the other hand, miR-373 functions *in trans* to recruit RNA polymerase II at the *E-cadherin* promoter to activate its expression in prostate cancer cells^45^. It has been indicated that miR-584, a recently identified tumor suppressive miRNA, is under-expressed in breast cancer^46^ and renal cell carcinoma^47^. However, the functions of miR-584-3p in gastric cancer still remain to be elucidated. In the current study, our findings showed that miR-584-3p recognized its binding site to repress the enrichment of YY1 on *MMP-14* promoter, resulting in decreased expression of MMP-14 in gastric cancer cells. The fact that ectopic expression of miR-584-3p was sufficient to prevent the gastric cancer cells from YY1-mediated biological behaviors indicates that the tumor suppressive functions of miR-584-3p are exerted, at least in part, through inhibiting the YY1 activity in gastric cancer.

Since AGO2 is crucial for miRNA-induced silent-state epigenetic markers at target promoters^21, 22^, we further observed the roles of AGO2 in miR-584-3p-mediated inhibition of MMP-14 expression in gastric cancer. We found that AGO2 was enriched surrounding the binding site of miR-584-3p within *MMP-14* promoter in gastric cancer cells. In addition, treatment of gastric cancer cells with RNase H, an enzyme degrading the RNA within RNA-DNA hybrid^21^, abolished the enrichment of AGO2 induced by miR-584-3p, indicating that miR-584-3p directly interacted with *MMP-14* promoter. Knockdown of *AGO2* abolished the miR-584-3p-induced binding of repressive epigenetic markers, accompanied by increased YY1 enrichment on *MMP-14* promoter. Previous studies indicate that YY1 acts as novel critical interface between epigenetic code and miRNAs machinery in suppressing gene expression^48^. However, in this study, our evidence indicated that YY1 served as a positive transcriptional regulator of MMP-14 expression, and was not associated with AGO2/miR-584-3p for histone methylation. We believe that miR-584-3p interacts with AGO2, which recruits EZH2 and EHMT2 to induce histone methylation and decrease the binding of YY1 to *MMP-14* promoter. Due to the efficiency of knockdown experiments, further investigation is warranted to explore whether the functions of miR-584-3p are dependent on AGO2 by using knockout cell lines.

In summary, we have shown that YY1 is highly expressed and directly binds to the *MMP-14* promoter to facilitate its transcription in gastric cancer. Furthermore, miR-584-3p is under-expressed in human gastric cancer, and suppresses the transcription of *MMP-14* via epigenetically suppressing the binding of YY1 to its promoter, resulting in decreased growth, invasion, metastasis, and angiogenesis of gastric cancer cells *in vitro* and *in vivo*. Our findings reveal the novel mechanisms of *MMP-14* gene expression associated with gastric cancer progression, and suggest that YY1 and miR-584-3p are potential targets for the therapeutics of gastric cancer. Meanwhile, the roles of YY1 and miR-584-3p in regulating the MMP-14 expression warrant further investigation in other types of cancers.

## Methods

### Cell line culture

Human gastric cancer cell lines [AGS (CRL-1739), SGC-7901, MKN-28, and MKN-45], prostate cancer cell line PC-3 (CRL-1435), colon cancer cell line LoVo (CCL-229), normal gastric epithelial GES-1 cells, and human endothelial cell line HUVEC (CRL-1730) were purchased from the American Type Culture Collection (Rockville, MD) and Type Culture Collection of Chinese Academy of Sciences (Shanghai, China). Cell lines were authenticated by the provider, and used within 6 months after resuscitation of frozen aliquots. Cancer cell lines were grown in RPMI1640 medium (Life Technologies, Inc., Gaithersburg, MD) containing 10% fetal bovine serum (Life Technologies, Inc.), penicillin (100 U/ml), and streptomycin (100 μg/ml). The HUVEC cell line was cultured in Ham’s F12K medium supplemented with 2 mmol/L L-glutamine, 0.1 mg/ml heparin, 0.03 mg/ml endothelial cell growth supplement (Millipore, Billerica, MA), and 10% fetal bovine serum (Life Technologies, Inc.), and used during passages 10 to 25. Cells were maintained at 37 °C in a humidified atmosphere of 5% CO_2_, and applied for transfection or treatment with C646, GSK343, or A-366 (Sigma, St. Louis, MO) as indicated.

### Over-expression and knockdown of genes

Human *YY1* expression construct pcDNA3-YY1 was kindly provided by Dr. Shourong Wu (Chongqing University, China)^49^. Human *MMP-14* expression vector was previously described^50^. The oligonucleotides encoding sh-Scb and shRNA specific for *YY1*, *p65*, *AGO2*, and *MMP-14* (Supplementary Table S4) were inserted into GV102 (Genechem Co., Ltd, Shanghai, China). Transfection of these vectors was performed using Lipofectamine 2000 (Invitrogen, Carlsbad, CA). After selecting for puromycin (Invitrogen) resistance, stable cell lines were obtained.

### Western blotting

Protein from cell lines and tissues was extracted using 1× cell lysis buffer (Promega, Madison, WI). The SDS-PAGE electrophoresis and immunoblotting were performed as previously described^51–54^, with antibodies specific for MMP-2 (Millipore), YY1, MMP-14, VEGF_165_, Snail, AGO2, and β-actin (Santa Cruz Biotechnology, Santa Cruz, CA).

### Real-time RT-PCR

Isolation of total RNA from cell lines and tissues was performed using RNeasy Mini Kit (Qiagen Inc., Valencia, CA). After the reverse transcription reactions with Transcriptor First Strand cDNA Synthesis Kit (Roche, Indianapolis, IN), real-time PCR was conducted using primers (Supplementary Table S5) and SYBR Green PCR Master Mix (Applied Biosystems, Foster City, CA). The transcript levels of genes were analyzed by 2^−ΔΔCt^ method.

### Prediction and measurement of miRNA

The algorithm microPIR^24^ was applied to analyze the potential miRNA targeting sites within *MMP-14* promoter. The miRNA-specific stem-loop primer, PCR primers (Supplementary Table S5), and Bulge-Loop^TM^ miRNAs qPCR Primer Set (RiboBio Co. Ltd, Guangzhou, China) were used to synthesize the cDNA and measure the levels of mature miR-584-3p. The results were analyzed by normalizing the miRNA levels to those of U6 snRNA.

### Over-expression and knockdown of miRNA

Based on the sequence in the miRNA Registry database^55^, the construct of miR-584-3p precursor was established by inserting the encoding oligonucleotides (Supplementary Table S4) into pcDNA3.1(−) (Invitrogen). After selecting for neomycin (Invitrogen) resistance, the miR-584-3p over-expressing stable cancer cell lines were obtained. To knockdown miR-584-3p, confluent cells were transfected with negative control or anti-miR-584-3p inhibitors (RiboBio Co. Ltd) using Lipofectamine 2000 (Invitrogen).

### Promoter activity assay

The luciferase reporter of human *MMP-14* promoter was a kind gift from Dr. Jouko Lohi^7^. The GeneTailor^TM^ Site-Directed Mutagenesis System (Invitrogen) and PCR primers (Supplementary Table S4) were applied to generate the constructs with mutant binding sites of YY1 or miR-584-3p. The activity of *MMP-14* promoter was measured by dual-luciferase assay^53, 54, 56^.

### Nascent transcription detection

The nascent transcription of genes within cancer cells was measured by nuclear run-on assay^56, 57^. After incorporation of biotin-16-uridine-5′-triphosphate, the Trizol and agarose-conjugated streptavidin beads (Invitrogen) were applied for extraction of total and biotinylated nascent RNA. Real-time RT-PCR was performed with primers (Supplementary Table S5) as above described.

### Chromatin immunoprecipitation assay

The EZ-ChIP kit (Upstate Biotechnology, Temacula, CA) was applied in ChIP assay^23, 53, 54, 56^, with antibodies specific for YY1, AGO2, EZH2, EHMT2, H3K27me3, and H3K9me2 (Upstate Biotechnology, Temacula, CA). Prior to immunoprecipitation, the RNase H (10 U) or RNase A (20 μg) was used to treat the lysates. The SYBR Green PCR Master Mix and primer sets (Supplementary Table S5) were applied for real-time qPCR. The input DNA, total amount of chromatin used in subsequent immunoprecipitation, was used as a control for normalization.

### Co-immunoprecipitation

Co-immunoprecipitation (co-IP) was performed as previously described^56^, with antibody specific for AGO2. The bead-bound protein was released by boiling the protein A-Sepharose beads (Santa Cruz Biotechnology) in 1× SDS-PAGE loading buffer and analyzed by western blot.

### Crosslink RNA immunoprecipitation (RIP)

Cells were ultraviolet light crosslinked at 254 nm (200 J/cm^2^) and collected by scraping. RIP assay was performed with AGO2 antibody as previously described^54^. The co-precipitated RNAs were detected by RT-PCR with specific primers (Supplementary Table S5). Total RNAs (input) and isotype antibody (IgG) were applied as controls.

### Cell viability, growth, and invasion assay

The MTT (Sigma) colorimetric^56^, soft agar^54, 58^, and matrigel invasion^23, 51–54, 59^ assays were performed to measure the *in vitro* viability, growth, and invasion capabilities of cancer cells.

### *In vitro* angiogenesis assay

The HUVEC cells were starved for 24 hrs, suspended in medium preconditioned with cancer cells, and added to the matrigel-coated 96-well plates (5 × 10^4^ cells/well). After incubation at 37 °C for 18 hrs, the angiogenic activity was detected and quantified^51, 58^.

### *In vivo* growth and metastasis assay

All animal experiments were carried out in accordance with NIH Guidelines for the Care and Use of Laboratory Animals, and approved by the Animal Care Committee of Tongji Medical College (approval number: Y20080290). The blindly randomized four-week-old male BALB/c nude mice (n = 5 for each group) were applied in the *in vivo* tumor growth and experimental metastasis studies^51–53^.

### Clinical specimens and measurement

Approval to conduct this study was obtained from the Institutional Review Board of Tongji Medical College (approval number: 2011-S085). All procedures were carried out in accordance with the approved guidelines. Informed consent was obtained from all of the patients. The fresh tumor and adjacent normal gastric specimens from 80 well-established primary gastric cancer cases were collected at surgery, validated by pathological diagnosis, stored at −80 °C, and used for detection of gene expression by western blotting and real-time RT-PCR. The demographic and clinicopathological details of subtotal 60 patients were indicated in Supplementary Table S1.

### Immunohistochemical staining

Immunohistochemical staining was undertaken as described previously^51–53^, with antibodies specific for YY1, MMP-14 (Santa Cruz Biotechnology; 1:200 dilutions), Ki-67, and CD31 (R&D Systems, Inc., Minneapolis, MN; 1:200 dilutions). The intratumoral Ki-67/CD31 ratio and necrosis score were evaluated as previously described^60^.

### Data availability

The datasets analyzed during the current study were available in the Gene Expression Omnibus (GEO; https://www.ncbi.nlm.nih.gov/geo/)^16–19, 28, 31, 32^ and ArrayExpress (http://www.ebi. ac.uk/arrayexpress/)^30^. All remaining data were contained within the article and supplementary information files or available from the author on request.

### Statistical analysis

All data were presented as mean ± standard error of the mean (SEM). To compare the gene expression and analyze the relationship among gene expression, the χ^2^ analysis, Fisher exact probability analysis, and Pearson’s coefficient correlation assay were applied. The log-rank test and Cox regression models were used to assess survival difference and hazard ratios. The *t* test and analysis of variance (ANOVA) were used to determine the difference of cancer cells.

## Electronic supplementary material

Supplementary Information
