# Supplementary material for: miRNA-584-3p inhibits gastric cancer progression by repressing Yin Yang 1- facilitated MMP-14 expression
Source: Sci Rep. 2017 Aug 21;7:8967. doi: 10.1038/s41598-017-09271-5 (PMC5566321; doi:10.1038/s41598-017-09271-5)
Supplement: Supplementary file 1 — Supplementary Information [file 41598_2017_9271_MOESM1_ESM.pdf]

miRNA-584-3p inhibits gastric cancer progression by repressing Yin Yang 1-facilitated MMP-14 expression

Liduan Zheng [1,2,#], Yajun Chen [1,#], Lin Ye [3,#], Wanju Jiao [1], Huajie Song [3], Hong Mei [3], Dan Li [3], Feng Yang [3], Huanhuan Li [3], Kai Huang [2], and Qiangsong Tong [2,3,*]

[1] Department of Pathology, Union Hospital, Tongji Medical College, Huazhong University of Science and Technology, 1277 Jiefang Avenue, Wuhan 430022, Hubei Province, P. R. China

[2] Clinical Center of Human Genomic Research, Union Hospital, Tongji Medical College, Huazhong University of Science and Technology, 1277 Jiefang Avenue, Wuhan 430022, Hubei Province, P. R. China

[3] Department of Surgery, Union Hospital, Tongji Medical College, Huazhong University of Science and Technology, 1277 Jiefang Avenue, Wuhan 430022, Hubei Province, P. R. China

Supplementary Figure Legends

Supplementary Figure S1 Effects of YY1 on MMP-14 expression. **a**, over-lapping analysis revealing YY1 as a crucial transcription factor of *MMP-14*. **b**, the correlation between *YY1* and *MMP-14* levels in public gastric cancer datasets derived from Gene Expression Omnibus (GEO). **c**, real-time quantitative RT-PCR showing the effects of YY1 and C646 on cellular *MMP-14* expression. * P<0.01 vs. mock.

Supplementary Figure S2 Expression of MMP-14 and VEGF in gastric cancer cells. Real-time quantitative RT-PCR indicating the *MMP-14* and *VEGF* levels in cancer cells with ectopic expression or knockdown of miR-584-3p (**a** and **b**), and those co-transfected with sh-Scb or sh-AGO2 (**c**). RIP and qPCR (**d**), western blot (**e**), and ChIP and qPCR (**f**) assays showing the interaction between miR-548-3p and AGO2, expression of H3K27me3 and H3K9me2, and protein

enrichment on *MMP-14* promoter in gastric cancer cells transfected with mock or miR-584-3p, and those co-transfected with sh-Scb, sh-AGO2, sh-YY1 or treated with GSK343 (1 μmol/L) or A-366 (3 μmol/L). * *P*<0.01 vs. mock, anti-NC, mock+sh-Scb, IgG, or mock+DMSO.

Supplementary Figure S3 Roles of YY1 and miR-584-3p in cancer cells. Real-time quantitative RT-PCR (**a**), western blot (**b**), dual-luciferase (**c**), ChIP (using YY1 antibody) and qPCR (**d**) assays showing the expression of MMP-14 and YY1, activity of *MMP-14* promoter reporter [pGL3-MMP14 (-1246/+199)], and YY1 binding to *MMP-14* promoter in cancer cells transfected with empty vector (mock) or miR-584-3p precursor, and those co-transfected with *YY1* (mean ± SD, n=4). **P*<0.01 vs. mock+mock.

Supplementary Figure S4 Effects of miR-584-3p and YY1 on gastric cancer cells. Western blot (**a**), MTT colorimetric (**b**), soft agar (**c**), matrigel invasion (**d**), and tube formation (**e**) assays indicating gene expression, viability, growth, invasion, and angiogenesis of cancer cells transfected with sh-Scb, sh-YY1, anti-NC, anti-miR-584-3p inhibitors (100 nmol/L). * *P*<0.01 vs. sh-Scb+anti-NC.

Supplementary Figure S5 miR-584-3p and YY1 exert functions through regulating MMP-14 expression. Western blot (**a** and **e**), MTT colorimetric (**b** and **f**), matrigel invasion (**c** and **g**), and tube formation (**d** and **h**) assays indicating gene expression, viability, invasion, and angiogenesis of cancer cells transfected as indicated. **P*<0.01 vs. mock+mock or mock+sh-Scb.

Supplementary Figure S6 Expression of miR-584-3p, SH3TC2, and MMP-14 in publicly available datasets. a and **b**, miR-584-3p and SH3TC2 levels in gastric cancer and adjacent normal

tissues derived from Gene Expression Omnibus (GEO) datasets. **c**, the correlation between SH3TC2 and MMP-14 levels in gastric cancer tissues derived from GEO datasets.

Supplementary Figure S7 Interaction between AGO2 with EZH2, EHMT2, and YY1 in gastric cancer cells. Co-IP and western blot assays showing the interaction of AGO2 with EZH2, EHMT2, and YY1 in MKN-45 cells stably transfected with mock or miR-584-3p precursor.

Supplementary Figure S1

a

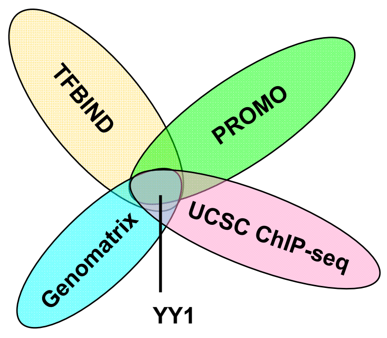

TF name	TF binding site within MMP-14 promoter region (chr14:23304582-23305827)			ChIP-seq peak (UCSC Browser)
	Genomatrix	TFBIND	PROMO	
YY1	Yes	Yes	Yes	Yes
NFY	Yes	Yes	Yes	No
NFE2L2	Yes	Yes	Yes	No

b

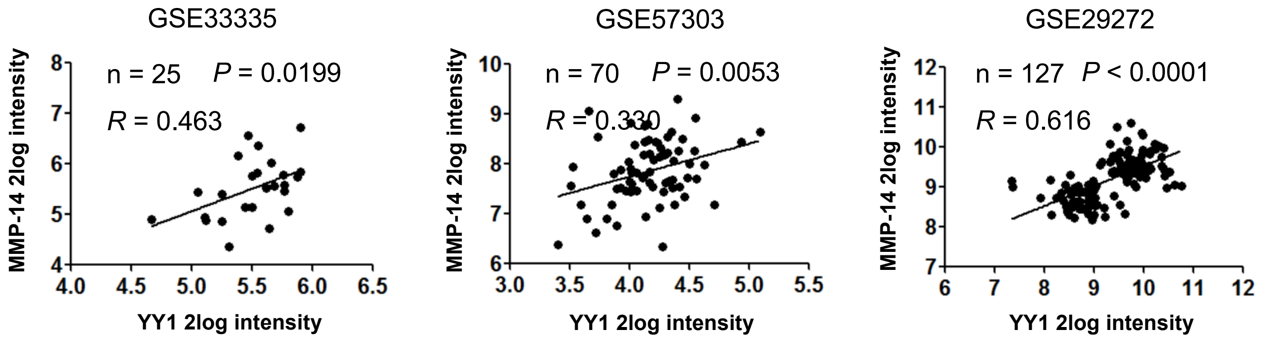

c

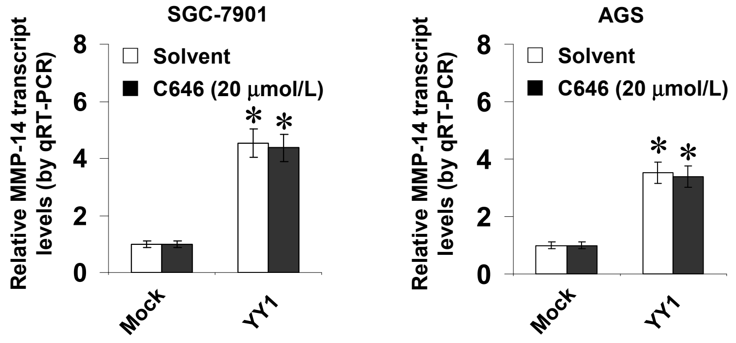

Supplementary Figure S2

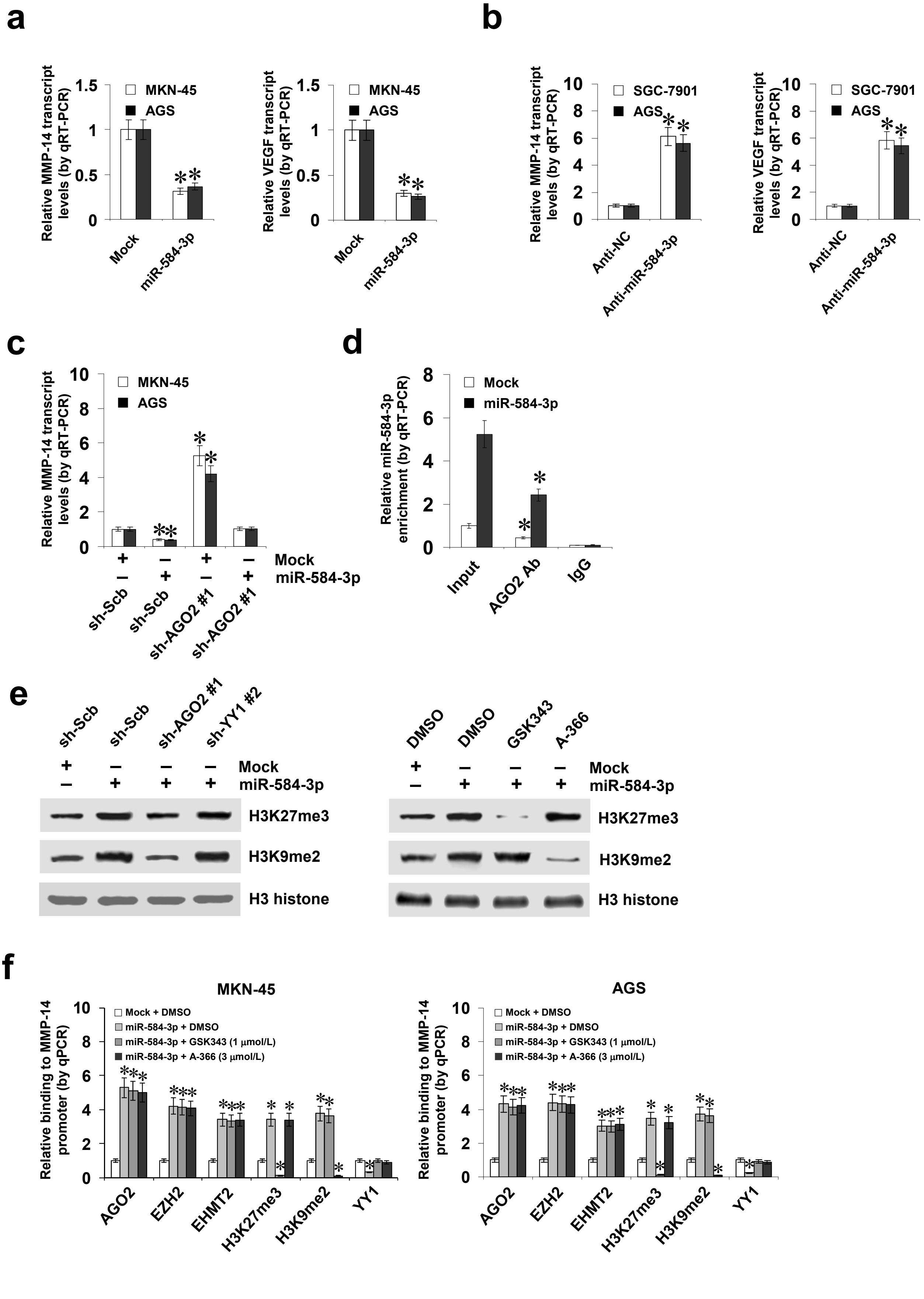

Supplementary Figure S3

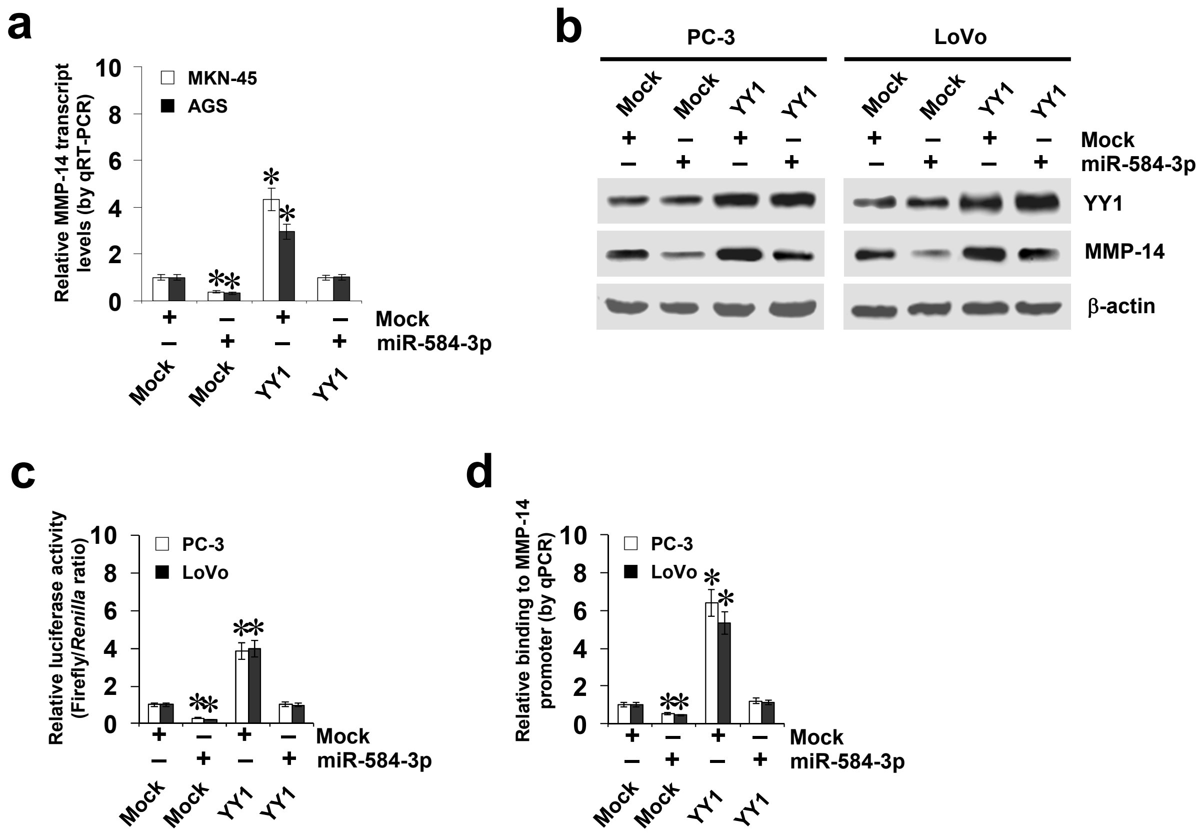

Supplementary Figure S4

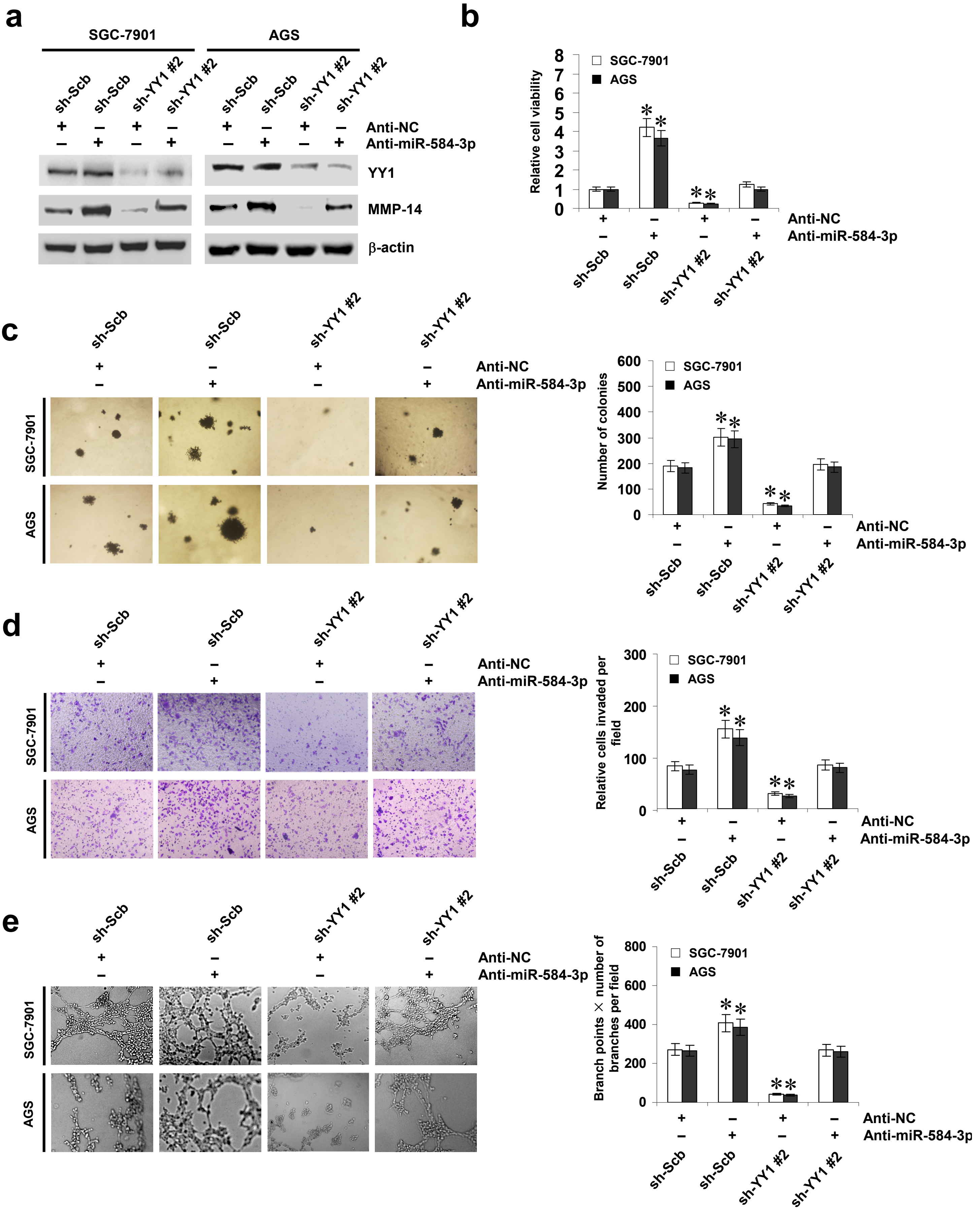

Supplementary Figure S5

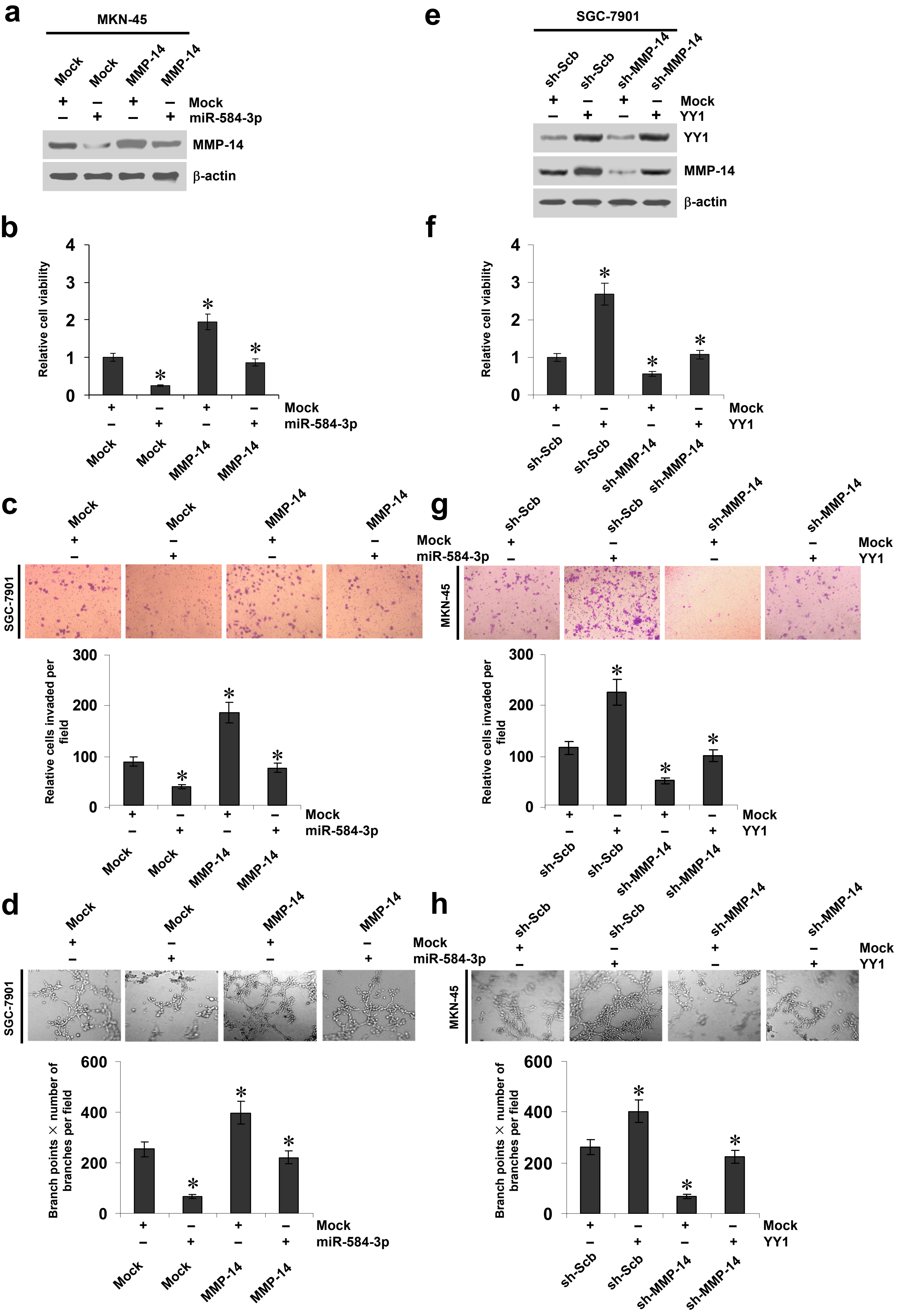

Supplementary Figure S6

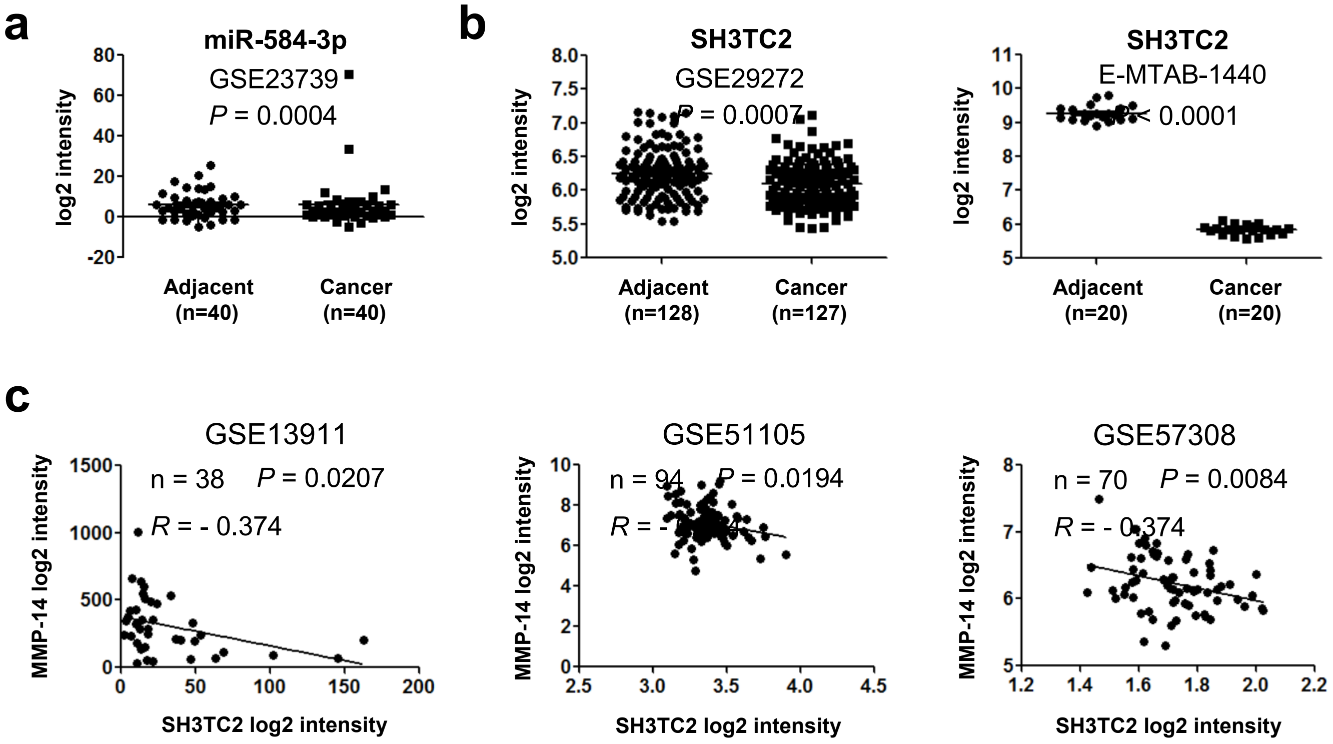

Supplementary Figure S7

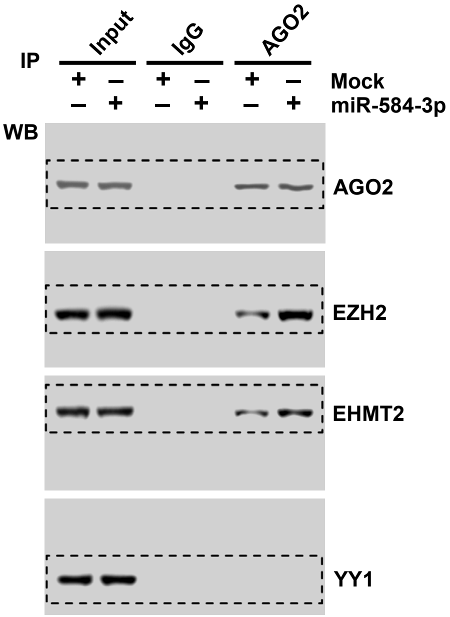

Supplementary Table S1 YY1 and MMP-14 expression in human gastric cancer tissues

Clinicopathologic factor	Total	YY1 expression		MMP-14 expression	
	n (%)	n (%)	*P*-value	n (%)	*P*-value
**Age (years)**					
⩽60	31 (51.7)	19 (61.3)	0.833	22 (70.9)	0.866
>60	29 (48.3)	17 (58.6)		20 (68.9)	
**Sex**					
Male	42 (70.0)	25 (59.5)	0.908	29 (69.0)	0.806
Female	18 (30.0)	11 (61.1)		13 (72.2)	
**Size (diameter)**					
⩽6 cm	40 (66.7)	24 (60.0)	1.000	28 (70.0)	1.000
>6 cm	20 (33.3)	12 (60.0)		14 (70.0)	
**Laurén classification**					
Intestinal type	35 (58.3)	20 (57.1)	0.593	24 (68.6)	0.775
Diffuse type	25 (41.7)	16 (64.0)		18 (72.0)	
**Gastric wall invasion**					
T1/T2	22 (36.7)	3 (13.6)	<0.001	8 (36.4)	<0.001
T3/T4	38 (63.3)	33 (86.8)		34 (89.5)	
**Lymph node metastasis**					
Negative	21 (35.0)	5 (23.8)	<0.001	6 (28.6)	<0.001
Positive	39 (65.0)	31 (79.5)		36 (92.3)	
**Distant metastasis**					
Negative	46 (76.7)	24 (52.2)	0.025	28 (60.9)	0.005
Positive	14 (23.3)	12 (85.7)		14 (100.0)	
**TNM stage**					
I/II	20 (33.3)	5 (25.0)	<0.001	6 (30.0)	<0.001
III/IV	40 (66.7)	31 (77.5)		36 (90.0)	

YY1, Yin Yang 1; MMP-14, matrix metalloproteinases 14; TNM, tumor-node-metastasis.

Supplementary Table S2 Correlation among the expression of YY1 and MMP-14

	YY1 expression			
	Low	High	*R*-value	*P*-value
**MMP-14 expression**				
Low	28	2	0.802	<0.001
High	4	26		

YY1, Yin Yang 1; MMP-14, matrix metalloproteinases 14; Pearson's correlation coefficient was applied to determine the expression correlation.

Supplementary Table S3 Univariate and multivariate analysis of prognostic factors in gastric cancer patients

Clinicopathologic factor	n	Univariate analysis		Multivariate analysis		
		Mean ± SEM (months)	*P*-value	Hazard ratio	95% CI	*P*-value
**Age (years)**						
≤60	31	33.2 ± 5.2	0.865	1.121	0.421-3.012	0.712
>60	29	35.1 ± 4.3				
**Sex**						
Male	42	34.4 ± 4.5	0.826	1.285	0.412-3.223	0.716
Female	18	33.5 ± 5.3				
**Size (diameter)**						
≤6 cm	40	34.1 ± 4.6	0.804	1.657	1.242-5.021	0.424
>6 cm	20	33.5 ± 6.2				
**Laurén classification**						
Intestinal type	35	39.3 ± 4.1	0.142	1.612	0.376-4.021	0.514
Diffuse type	25	30.1 ± 5.2				
**Gastric wall invasion**						
T1/T2	22	61.5 ± 0.6	<0.001	2.134	1.455-6.252	0.446
T3/T4	38	19.5 ± 2.4				
**Lymph node metastasis**						
Negative	21	60.2 ± 0.8	<0.001	2.018	1.212-5.323	0.523
Positive	39	21.5 ± 2.2				
**Distant metastasis**						
Negative	46	42.9 ± 3.2	<0.001	2.126	1.854-5.421	0.003
Positive	14	9.8 ± 0.7				
**TNM stage**						
I/II	20	62.1 ± 0.9	<0.001	2.352	1.698-6.124	0.001
III/IV	40	21.4 ± 2.7				
**miR-584-3p expression**						
Low	31	18.5 ± 2.7	<0.001	0.312	0.131-0.765	0.018
High	29	55.7 ± 2.5				
**YY1 expression**						
Low	32	54.5 ± 3.1	<0.001	2.743	1.215-5.236	0.011
High	28	18.1 ± 2.3				

YY1, Yin Yang 1; TNM, tumor-node-metastasis. Log-rank test and Cox regression model were applied for univariate and multivariate analysis. *n*, number of patients; SEM, standard error of the mean; 95% CI, 95% confidence interval.

Supplementary Table S4 Oligonucleotide sets used for constructs, inhibitors and short hairpin RNAs

Oligo Set	Sequences
Pre-miR-584-3p	5'-TGCTGTTATGGTTTGCCTGGGACTGAGGTTTTGGCCACTGACTGACCTCAGTCCCAGGCAAACCATAA-3' (sense); 5'-CCTGTTATGGTTTGCCTGGGACTGAGGTCAGTCAGTGGCCAAAACCTCAGTCCCAGGCAAACCATAAC-3' (antisense)
Pre-miR-NC	5'-TGCTGAAATGTACTGCGCGTGGAGACGTTTTGGCCACTGACTGACGTCTCCACGCAGTACATTT-3' (sense); 5'-CCTGAAATGTACTGCGTGGAGACGTCAGTCAGTGGCCAAAACGTCTCCACGCGCAGTACATTTC-3' (antisense)
pGL3-MMP14 mut (ΔYY1)	5'-CAACCCCTGCGTGCAATAGCCTGCACCACAAAAAGGCAAC TTAGAGGT-3' (sense); 5'-TGGTGCAGGCTATTGCACGCAGGGGTTGAGGCCGCGATGGACAGCAGG-3' (antisense)
pGL3-MMP14 mut (ΔmiR-584-3p)	5'-CATCCCGTCCAAGTTAGCCAACTACGGCCCCCTGCTGTCCATCGCGGCCTCAACCCC-3' (sense); 5'-CAGCAGGGGGCCGTAGTTGGCTAACTTGGACGGGATGTGGGAGACTTTGTCCCTATA-3' (antisense)
Anti-NC	RiboBio
Anti-miR-584-3p	RiboBio
sh-Scb	5'-CCGGGCGAACGATCGAGTAAACGGACTCGAGTCCGTTTACTCGATCGTTCGCTTTTT-3' (sense); 5'-AATTCAAAAAGCGAACGATCGAGTAAACGGACTCGAGTCCGTTTACTCGATCGTTCGC-3' (antisense)
sh-YY1 #1	5'-CCGGCGATGGTTGTAATAAGAAGTTCTCGAGAACTTCTTATTACAACCATCGTTTTTG-3' (sense); 5'-AATTCAAAAACGATGGTTGTAATAAGAAGTTCTCGAGAACTTCTTATTACAACCATCG-3' (antisense)
sh-YY1 #2	5'-CCGGCCCAAACAACTGGCAGAATTTCTCGAGAAATTCTGCCAGTTGTTTGGGTTTTTG-3' (sense); 5'-AATTCAAAAACCCAAACAACTGGCAGAATTTCTCGAGAAATTCTGCCAGTTGTTTGGG-3' (antisense)
sh-p65 #1	5'-CCGGGCCTTAATAGTAGGGTAAGTTCTCGAGAACTTACCCTACTATTAAGGCTTTTTG-3' (sense); 5'-AATTCAAAAAGCCTTAATAGTAGGGTAAGTTCTCGAGAACTTACCCTACTATTAAGGC-3' (antisense)
sh-p65 #2	5'-CCGGCGGATTGAGGAGAAACGTAAACTCGAGTTTACGTTTCTCCTCAATCCGTTTTTG-3' (sense); 5'-AATTCAAAAACGGATTGAGGAGAAACGTAAACTCGAGTTTACGTTTCTCCTCAATCCG-3' (antisense)
sh-AGO2 #1	5'-CCGGGCACAGCCAGTAATCGAGTTTCTCGAGAAACTCGATTACTGGCTGTGCTTTTTG-3' (sense); 5'-AATTCAAAAAGCACAGCCAGTAATCGAGTTTCTCGAGAAACTCGATTACTGGCTGTGC-3' (antisense)
sh-AGO2 #2	5'-CCGGCGTCCGTGAATTTGGAATCATCTCGAGATGATTCCAAATTCACGGACGTTTTTG-3' (sense); 5'-AATTCAAAAACGTCCGTGAATTTGGAATCATCTCGAGATGATTCCAAATTCACGGACG-3' (antisense)
sh-MMP-14	5'-CCGGCGCCAGAAGCTGAAGGTAGAACTCGAGTTCTACCTTCAGCTTCTGGCGTTTTTG-3' (sense); 5'-AATTCAAAAACGCCAGAAGCTGAAGGTAGAACTCGAGTTCTACCTTCAGCTTCTGGCG-3' (antisense)

Pre-miR-NC, negative control pre-miRNA; MMP-14, matrix metalloproteinases 14; Anti-NC, negative control inhibitor; sh-Scb, scramble short hairpin RNA; YY1, Yin Yang 1; AGO1, argonaute 1; AGO2, argonaute 2.

Supplementary Table S5 Primer sets used for qRT-PCR, nuclear run-on, RIP and ChIP

Primer set	Primers	Sequence	Product size (bp)	Application
MMP-14	Forward	5'-GCCTTCTGTTCCTGATAA-3'	225	qRT-PCR
	Reverse	5'-CCATCCTTCCTCTCGTAG-3‘		nuclear run-on
VEGF	Forward	5'-ATGACGAGGGCCTGGAGTGT-3'	226	qRT-PCR
	Reverse	5'-CATTTACACGTCTGCGGATCT-3'		
YY1	Forward	5'-GTGCAGAATGTGGCAAAGC-3'	168	qRT-PCR
	Reverse	5'-AGGGCCTGTCTCCGGTATG-3'		
β-actin	Forward	5'-ATCTACGAGGGGTATGCC-3'	227	qRT-PCR
	Reverse	5'-TAGCTCTTCTCCAGGGAG-3'		
miR-584-3p	Forward	RiboBio		qRT-PCR, RIP
	Reverse	RiboBio		
U6	Forward	RiboBio		qRT-PCR
	Reverse	RiboBio		
MMP-14 set 1	Forward	5'-TCAAGCCACTCAGAATATGC-3'	197	ChIP
(-326/-130)	Reverse	5'-GGTTGTTTTAGCCTGAATCC-3'		
MMP-14 set 2	Forward	5'-CAACCAGGAAAGGAGGGC-3'	191	ChIP
(-122/+69)	Reverse	5'-TCGGCTTGGAGTTAAAGG-3'		

MMP-14, matrix metalloproteinases 14; VEGF, vascular endothelial growth factor; YY1, Yin Yang 1.
